# Estrogen Modulates NFκB Signaling by Enhancing IκBα Levels and Blocking p65 Binding at the Promoters of Inflammatory Genes via Estrogen Receptor-β

**DOI:** 10.1371/journal.pone.0036890

**Published:** 2012-06-19

**Authors:** Dongqi Xing, Suzanne Oparil, Hao Yu, Kaizheng Gong, Wenguang Feng, Jonathan Black, Yiu-Fai Chen, Susan Nozell

**Affiliations:** 1 Vascular Biology and Hypertension Program, Division of Cardiovascular Disease, Department of Medicine, University of Alabama at Birmingham, Birmingham, Alabama, United States of America; 2 Department of Cell Biology, University of Alabama at Birmingham, Birmingham, Alabama, United States of America; 3 Department of Cardiology, The Second Clinical Medical School, Yangzhou University, Yangzhou, China; Oklahoma Medical Research Foundation, United States of America

## Abstract

**Background:**

NFκB signaling is critical for expression of genes involved in the vascular injury response. We have shown that estrogen (17β-estradiol, E2) inhibits expression of these genes in an estrogen receptor (ER)-dependent manner in injured rat carotid arteries and in tumor necrosis factor (TNF)-α treated rat aortic smooth muscle cells (RASMCs). This study tested whether E2 inhibits NFκB signaling in RASMCs and defined the mechanisms.

**Methodology/Principal Findings:**

TNF-α treated RASMCs demonstrated rapid degradation of IκBα (10–30 min), followed by dramatic increases in IκBα mRNA and protein synthesis (40–60 min). E2 enhanced TNF-α induced IκBα synthesis without affecting IκBα degradation. Chromatin immunoprecipitation (ChIP) assays revealed that E2 pretreatment both enhanced TNF-α induced binding of NFκB p65 to the *IκBα* promoter and suppressed TNF-α induced binding of NFκB p65 to and reduced the levels of acetylated histone 3 at promoters of *monocyte chemotactic protein (MCP)-1* and *cytokine-induced neutrophil chemoattractant (CINC)-2β* genes. ChIP analyses also demonstrated that ERβ can be recruited to the promoters of *MCP-1* and *CINC-2β* during co-treatment with TNF-α and E2.

**Conclusions:**

These data demonstrate that E2 inhibits inflammation in RASMCs by two distinct mechanisms: promoting new synthesis of IκBα, thus accelerating a negative feedback loop in NFκB signaling, and directly inhibiting binding of NFκB to the promoters of inflammatory genes. This first demonstration of multifaceted modulation of NFκB signaling by E2 may represent a novel mechanism by which E2 protects the vasculature against inflammatory injury.

## Introduction

Inflammation plays a major role in the pathogenesis of vascular disease [1]–[7]. Medial smooth muscle cells (SMCs) are critical target cells that are activated in the early phase of the vascular injury response and signal to other cells, i.e. monocytes, neutrophils, and adventitial fibroblasts, as well as to other SMCs, in orchestrating subsequent vascular remodeling [8]–[12]. In vitro, SMCs respond to pro-inflammatory stimuli, e.g. tumor necrosis factor (TNF)-α with increased expression of chemokines, cytokines and adhesion factors, thus promoting an inflammatory response. In the setting of acute endoluminal injury, 17β-estradiol (E2) inhibits inflammatory cytokine and chemokine expression, monocyte and neutrophil infiltration and neointima formation in carotid arteries of ovariectomized rats via an estrogen receptor (ER) dependent mechanism [8]–[10], [13]–[15]. Additionally, we have shown that in vitro, E2 inhibits TNF-α induced inflammatory mediator expression in isolated rat aortic (RA) SMCs in an ERβ-dependent manner [16].

In the setting of vascular injury, TNF-α activates NFκB, a transcription factor that mediates the immediate-early inflammatory response [17]–[20]. Although numerous NFκB proteins exist, the most common NFκB heterodimer contains p65 and p50. Each of the NFκB proteins contains an N-terminal Rel homology domain (RHD), which is important for DNA binding, dimerization, inhibitor association and nuclear localization [21], [22]. In most cells, NFκB is bound to and inhibited by IκBα, which reduces the ability of NFκB to bind DNA [23]. In response to TNF-α, interleukin-1β (IL-1β), or other stimuli, the inhibitor of NFκB kinase (IKK) complex is activated and phosphorylates IκBα, which targets it for degradation by the proteasome. This effectively liberates NFκB, which then translocates into the nucleus where it binds to cognate DNA response elements found within the promoters of target genes to induce their expression. NFκB activation is critical for the expression of a variety of genes, including *IκBα* and those involved in vascular inflammation, e.g. *cytokine-induced neutrophil chemoattractant (CINC)-2β* and *monocyte chemotactic protein (MCP)-1* [24]–[26]. Previously, we have shown that expression of MCP-1 and CINC-2β is inhibited by E2 in an ER dependent manner in balloon injured carotid arteries of rats and in RASMCs in vitro [9], [16]. However, at present, it is not clear exactly how E2 inhibits NFκB mediated expression of these genes in SMCs. The current study tested directly the hypothesis that E2, in an ER dependent manner, modulates the inflammatory response to TNF-α stimulation in isolated RASMCs in vitro by interfering with NFκB signaling and defined the precise sites of molecular merging of E2 and NFκB signaling cascades that are responsible for this effect.

## Results

### E2 does not Prevent IκBα Phosphorylation and Degradation, but does Enhance IκBα mRNA and Protein Levels in TNF-α treated RASMCs

**Figure 1:**
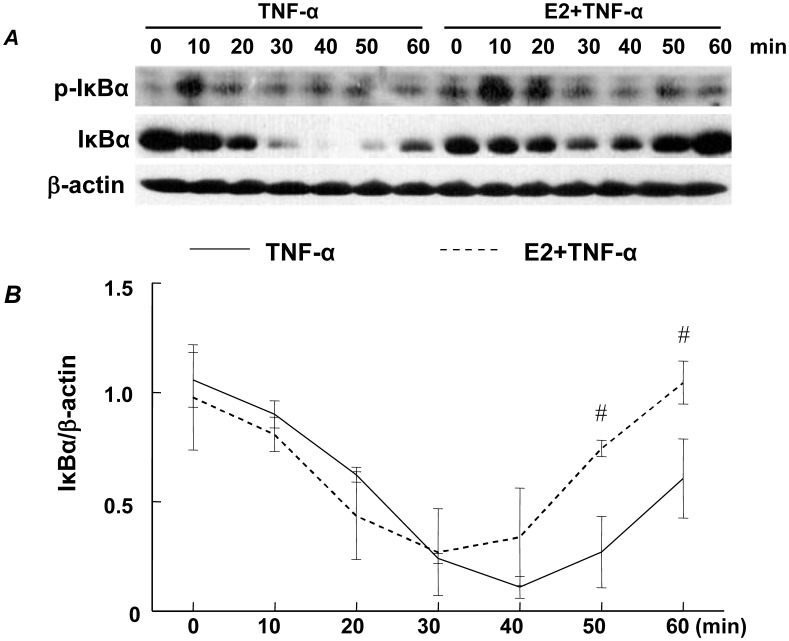
Representative Western blots of phospho-IκBα and IκBα in E2±TNF-α treated RASMCs. Cells were pretreated with/without E2 (10^−^7 M) for 24 hrs then stimulated with TNF-α (1 ng/mL) for the times shown (A). Line graph shows the ratio of IκBα to β-actin in E2±TNF-α treated RASMCs (B). Results are mean±SE from 3 samples/group. #p<0.05 vs. TNF-α-treated RASMCs.

Consistent with previous observations that IκBα processing is a target for E2/ER signaling [27]–[29], we tested the hypothesis that E2 inhibits cytokine-induced IκBα phosphorylation and degradation in RASMCs, thus attenuating NFκB signaling. Quiescent RASMCs were incubated with E2 or vehicle for 24 hrs, followed by TNF-α for 10, 20, 30, 40, 50 and 60 mins. Total protein was extracted and the levels of total and phospho-IκBα were assessed using Western blot analyses. RASMCs treated with TNF-α for 10 min demonstrated increased levels of phospho-IκBα, with rapid degradation of IκBα between 10–30 min (Figure 1A), followed by a dramatic recovery at 60 min. Levels of phospho-IκBα were not reduced by pretreatment with E2 (Figure 1). Although IκBα was degraded in the presence of E2 and TNF-α between 10–30 min, the total levels of IκBα were elevated compared to those in the presence of TNF-α alone between 30–60 min (Figure 1A). These results were analyzed by densitometry and are presented in Figure 1B. Because E2 does not prevent TNF-α induced IκBα degradation, these data suggest that E2 may attenuate NFκB signaling by inducing new IκBα mRNA synthesis.

**Figure 2:**
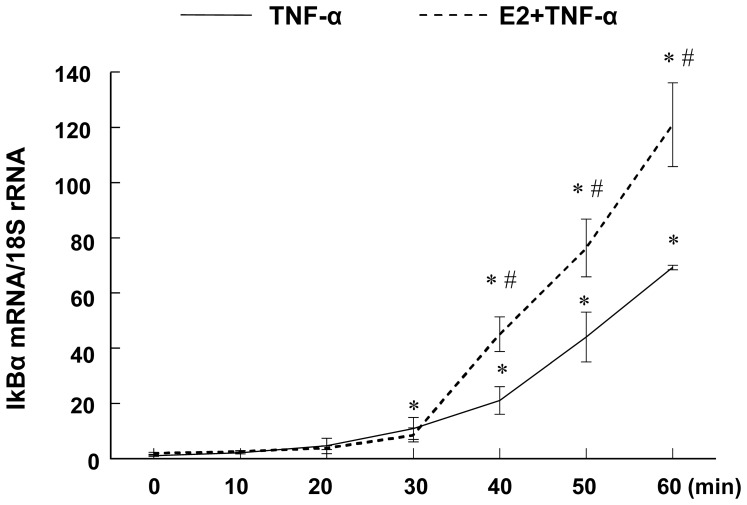
IκBα mRNA expression measured by real-time RT-PCR and normalized using 18 S rRNA. Cells were pretreated with/without E2 (10^−7^ M) for 24 hrs then stimulated with TNF-α (1 ng/mL) for the times shown. Results are mean±SEM from 6 wells/group. *p<0.05 vs. Vehicle-treated RASMCs; #p<0.05 vs. TNF-α-treated RASMCs.

To evaluate the effects of E2 on TNF-α induced IκBα mRNA levels, RASMCs were treated as described above and IκBα levels were analyzed using real-time RT-PCR analyses. The levels of IκBα mRNA were increased by TNF-α stimulation between 30–60 min (Figure 2), and were further enhanced by E2. These findings suggest that E2 can reduce NFκB activity by increasing the expression of IκBα mRNA and protein.

### ERβ Activation Enhances IκBα mRNA Expression and Restoration of IκBα Protein in TNF-α treated RASMCs

We have previously shown that in vitro, E2 inhibits TNF-α induced inflammatory mediator expression in RASMCs in an ERβ-dependent manner [16]. To test whether the effects of E2 on TNF-α-induced IκBα expression are also mediated by ERβ, RASMCS were pretreated with the selective ERβ agonist diarylpropiolnitrile (DPN), the selective ERα antagonist methyl-piperidinopyrazole (MPP) alone or in combination with E2, E2 alone or vehicle for 24 hrs, followed by TNF-α for an additional 45 or 60 min and subjected to Western blot analysis for IκBα protein and real-time RT-PCR analysis for IκBα mRNA, respectively. These time points were chosen because they capture the recovery phase of IκBα resynthesis following TNF-α induced phosphorylation and degradation (Figures 1 and 2).

**Figure 3:**
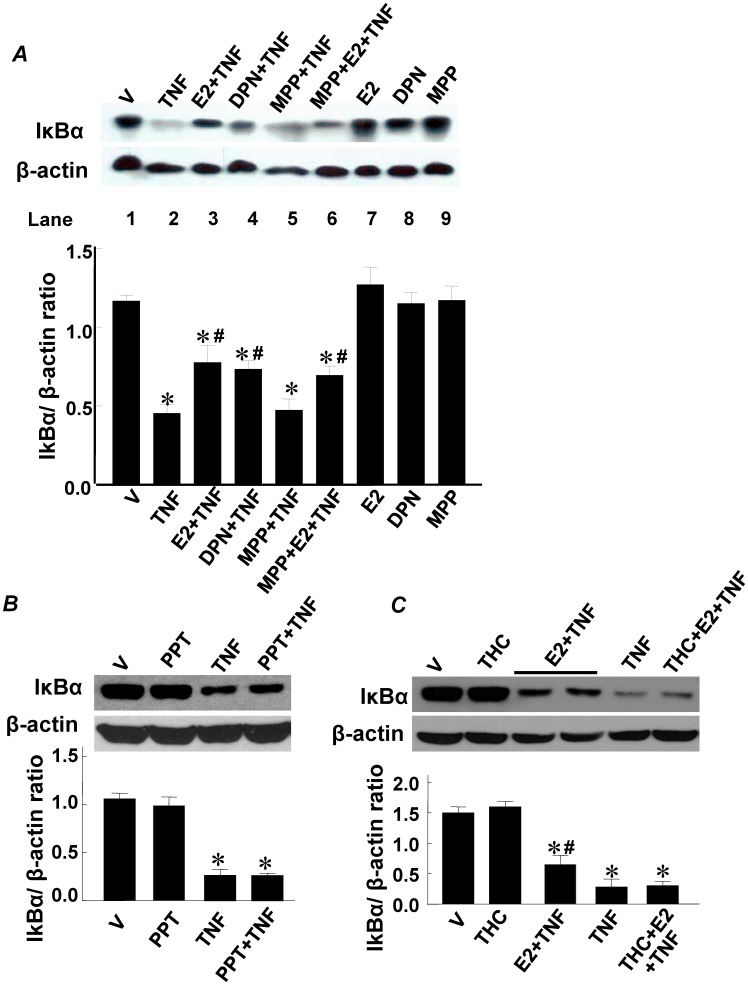
Role of ER isoforms on IκBα protein level. A. Pretreatment of ERβ agonist DPN (10^−7^ M) or E2 enhanced IκBα protein level in response to TNF-α treatment compared to TNF-α alone; ERα antagonist MMP (10^−6^ M) did not block the effect of E2 in TNF-α-treated cells. B. Pretreatment of ERα agonist PPT (10^−7^ M) did not affect IκBα protein level in response to TNF-α treatment compared to TNF-α alone; C. ERβ antagonist R,R- THC (10^−6^ M) blocked the effect of E2 in TNF-α-treated cells. Cells were pretreated with E2, DPN, PPT or vehicle for 24 h, then treated with TNF-α (1 ng/ml) for an additional 45 min. In some experiment groups, cells were pretreated with THC or MPP for 1 hr prior of E2. Bar graph shows the densitometric analysis of relative IκBα expression normalized to to β-actin Level. Results are mean±SE from 6 samples/group. *p<0.05 vs. Vehicle-treated RASMCs; #p<0.05 vs. TNF-α-treated RASMCs.

At 45 min post TNF-α treatment, IκBα protein levels were significantly lower in TNF-α treated RASMCs than in vehicle-treated control cells (Figure 3A, lane 2), indicating that IκBα protein expression had not completely recovered to vehicle control levels (lane 1) at this time point Pretreatment with E2 or DPN for 24 hr significantly accelerated the recovery of IκBα protein levels in TNF-α-treated cells (lanes 3, 4). In contrast, pretreatment with the ER α agonist propylpyrazole triol (PPT) did not alter the inhibitory effect of TNF-α on IκBα protein levels (Figure 3B, lane 4). In addition, the stimulatory effect of E2 on IκBα protein levels in TNF-α-treated cells was abolished by pretreatment with tetrahydrochrysene-R,R,-enantiomer (R,R-THC, an agonist on ERα and an antagonist on ERβ) 1 hr prior of E2 (Figure 3C, lane 6), but was not affected by pretreatment with the ERα antagonist MPP (Figure 3A, lane 6), supporting the ERβ dependency of the effect. E2, DPN, MPP (Figure 3A, lanes 7, 8, 9), PPT (Figure 3B, lane 2), or R,R-THC (Figure 3C, lane 2) alone had no effect on IκBα protein levels in RASMCs. These results provide evidence that the effect of E2 on TNF-α-regulated IκBα protein expression is mediated by ERβ, not ERα.

**Figure 4:**
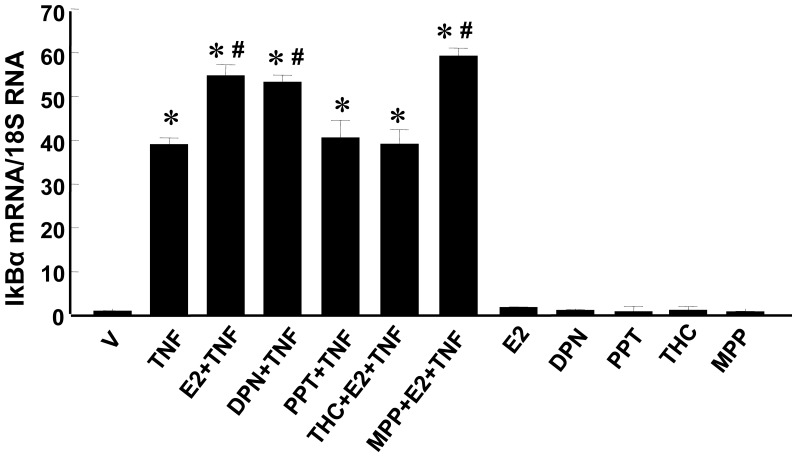
IκBα mRNA expression measured by real-time RT-PCR and normalized using 18 S rRNA. Cells were pretreated with E2 (10^−7^ M), DPN (10^−7^ M), PPT (10^−7^ M) or vehicle for 24 hr, then treated with TNF-α (1 ng/ml) for an additional 1 hr. MPP (10^−6^ M) or THC (10^−6^ M) was given to cells at 1 h before E2 treatment in some experiments. Results are mean±SEM from 6–9 wells/group. *p<0.05 vs. Vehicle-treated RASMCs; #p<0.05 vs. TNF-α-treated RASMCs.

Quantitative real time RT-PCR analysis demonstrated that IκBα mRNA levels were significantly increased in RASMCs at 60 min post TNF-α treatment (Figure 4, lane 2) compared to the vehicle control. Pretreatment with E2 or DPN (lanes 3 and 4), but not PPT (lane 5), further increased IκBα mRNA levels in TNF-α-treated RASMCs. The stimulatory effect of E2 on IκBα mRNA expression in TNF-α-treated cells was blocked by R,R-THC (lane 6), but not MPP (lane 7). E2, DPN, PPT, MPP or R,R-THC alone (lanes 8–12) did not alter IκBα mRNA levels in RASMCs in the absence of TNF-α treatment. Together, these findings suggest that the E2 mediated enhancement of IκBα mRNA expression in TNF-α treated RASMCs is mediated by ERβ, not ERα.

### E2, Through ERβ, Recruits NFκB p65 to the *IκBα* Promoter

**Figure 5:**
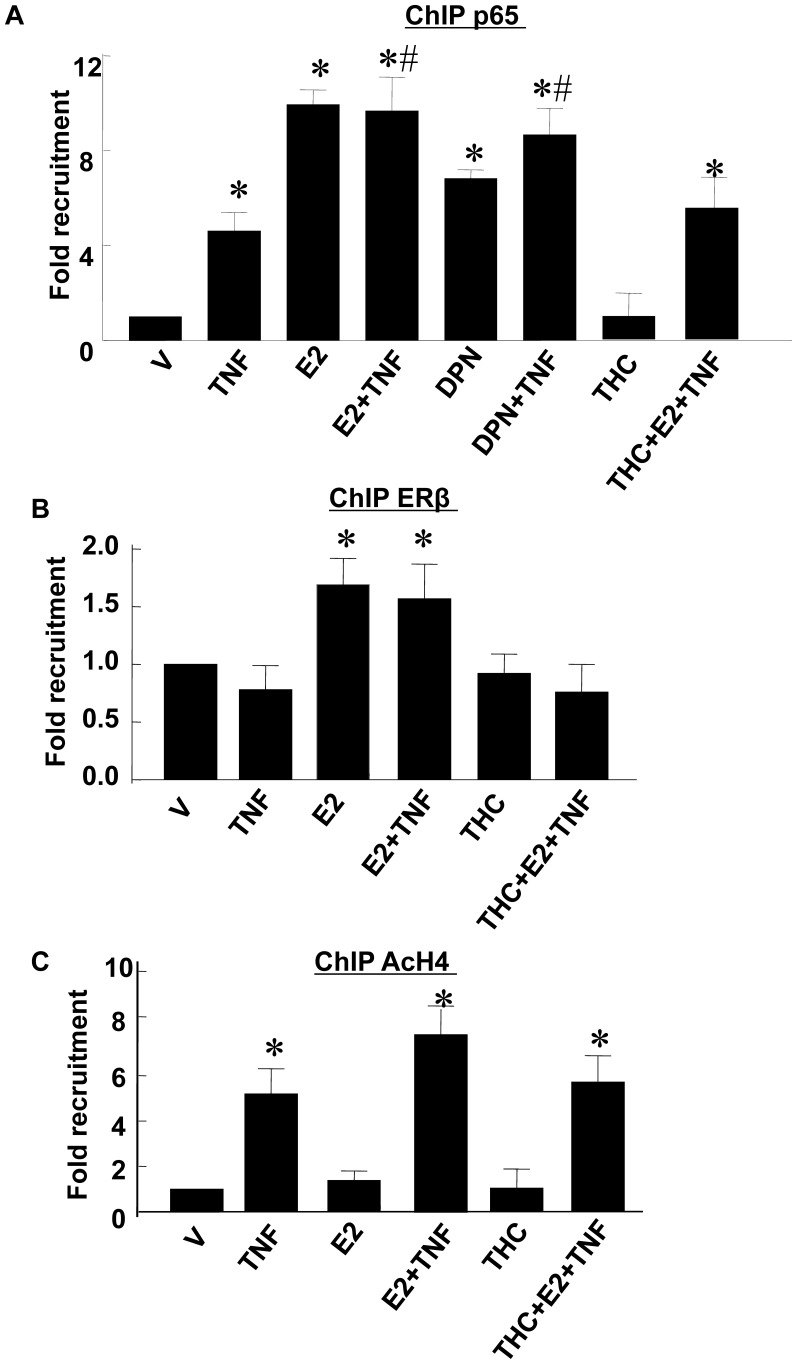
ChIP assays of the binding of NFκB p65 (A), ERβ (B) and AcH4 (C) to the *IκBα* promoter. Cells were pretreated with/without E2 (10^−7^ M) or DPN (10^−7^ M) for 24 hrs and then stimulated with TNF-α (1 ng/mL) for 1 hr. THC (10^−6^ M) was given to cells at 1 h before E2 treatment in some experiments. ChIP samples were prepared as described in the text and analyzed using antibodies specific for p65, ERβ or AcH4. The immunoprecipitated DNA fragments and input DNA were analyzed by real-time PCR. The y axis shows values were normalized to input DNA with values for vehicle treatment defined as 1. The numbers represent the mean±SEM from three experiments repeated in duplicate. *p<0.05 vs. Vehicle-treated RASMCs; #p<0.05 vs. TNF-α-treated RASMCs.

To understand the molecular mechanisms by which E2 might enhance IκBα mRNA synthesis, Chromatin Immunoprecipitation (ChIP) analyses were performed. Quiescent cells were pretreated with E2, DPN or vehicle for 24 hrs and then treated with TNF-α for 1 hr. In vehicle treated cells, ChIP assays revealed that NFκB p65 was not detected at the *IκBα* promoter (Figure 5, lane 1). Treatment with TNF-α, E2 or DPN alone (lanes 2, 3 and 5) resulted in recruitment of p65 (4 to 9 fold) to the *IκBα* promoter compared to vehicle control. When cells were pretreated with E2 or DPN and then challenged with TNF-α (lanes 4 and 6), the levels of p65 at the *IκBα* promoter were not altered significantly in response to additional TNF-α compared to the levels in the presence of E2 or DPN alone. In addition, pretreatment with the ERβ antagonist R,R-THC blocked E2 induced recruitment of p65 to the *IκBα* promoter in TNF-α-treated cells (lane 8), indicating ERβ dependency of the effect.

ChIP analyses with anti-ERβ antibody were performed to test whether ERβ was recruited to the *IκBα* promoter. In the vehicle treated cells (Figure 5B, lane 1), ERβ was detectable at the *IκBα* promoter. TNF-α treatment did not alter the binding of ERβ at the *IκBα* promoter (lane 2). In the E2 alone or E2+TNF-α treated cells, ERβ level was increased 2-fold at the *IκBα* promoter (lanes 3 and 4). E2 induced-recruitment of ERβ to the *IκBα* promoter was abolished by pretreatment with the ERβ antagonist R,R-THC (lane 5). In contrast, ERα was not detected at the *IκBα* promoter in response to E2 alone or coincides with the increased level of p65 at the *IκBα* promoter in the presence of E2 or E2+TNF-α (Data not shown).

Histones are acetylated at promoters that are undergoing active transcription [30]. The binding of acetylated histone at the promoter of a gene indicates that the gene is actively transcribing. ChIP assays determined that the levels of AcH4 at the *IκBα* promoter increased 5-fold in response to TNF-α treatment compared to vehicle (Figure 5C, lanes 1 and 2). E2 alone had no effect on binding of AcH4 to the *IκBα* promoter (lane 3). In the presence of E2+TNF-α, the levels of AcH4 at the *IκBα* promoter increased significantly (7-fold) compared to vehicle treatment (lane 4). The level of AcH4 at the *IκBα* promoter in the presence of E2+TNF-α was higher (about 40%) than the level in the presence of TNF-α alone, but the difference was not statistically significant. In cells pretreated of R,R-THC prior to E2+TNF-α (lane 6), the level of AcH4 at the *IκBα* promoter was not significantly different from the levels in E2+TNF-α treated cells. Together, these data suggest that treatment with E2, combined with TNF-α, significantly enhanced the transcriptional activity of the *IκBα* gene through an effect on ERβ.

### E2, Through ERβ, Inhibits the Binding of NFκB to the *MCP-1* and *CINC-2*β Promoters

**Figure 6:**
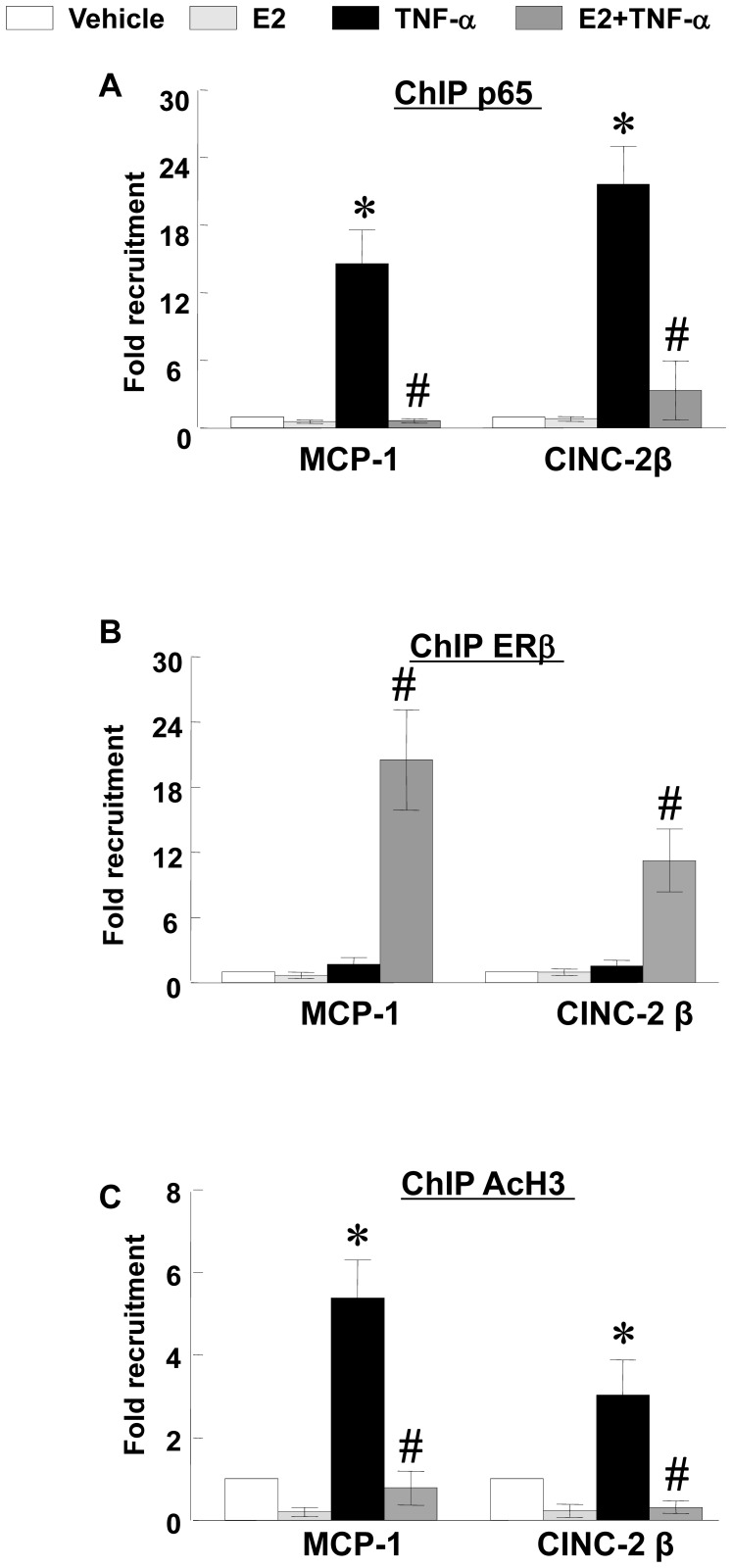
ChIP assays of binding of NFκB p65, ERβ and AcH3 to the *MCP-1* and *CINC-2β* promoters. Cells were pretreated without or with E2 for 24 hrs, then stimulated with TNF-α (1 ng/mL) for 1 hr. ChIP samples were prepared as described in the text and analyzed using antibodies specific for p65, ERβ or AcH3. The immunoprecipitated DNA fragments and input DNA were analyzed by by real-time PCR. The y axis shows values were normalized to input DNA with values for vehicle treatment defined as 1. The numbers represent result from three experiments repeated in duplicate. *p<0.05 vs. Vehicle-treated RASMCs; #p<0.05 vs. TNF-α-treated RASMCs.

ChIP assays determined that NFκB p65 was present at the *MCP-1* and *CINC-2*β promoters at low levels in the absence of TNF-α or E2 (Figure 6A) and that these levels were not affected by addition of E2 alone. At 1 hr post TNF-α treatment, the levels of NFκB p65 at these promoters were increased (14- and 21-fold), and these levels were reduced nearly to the control levels in the presence of pretreatment with E2, suggesting that E2 inhibits the ability of NFκB p65 to bind the promoters of these genes.

In the absence of TNF-α or E2 (Figure 6B), or in the presence of E2 alone or TNF-α alone, ERβ was barely detected at the *MCP-1* or *CINC-2*β promoters. However, in the presence of E2+TNF-α, ERβ was detected at the *MCP-1* and *CINC-2*β promoters. These data suggest that in the presence of E2+TNF-α, ERβ is recruited to these promoters and that the presence of ERβ coincides with the reduced levels of NFκB p65.

ChIP assays determined that the *MCP-1* and *CINC-2β* promoters harbored moderate levels of AcH3 in the absence of any stimuli (Figure 6C,), and that these levels were reduced in the presence of E2 alone. TNF-α treatment increased the levels of AcH3 at both promoters (5 and 3 fold, respectively) and these levels were diminished in the presence of E2, indicating that these genes have reduced transcriptional activity in the presence of E2. Together, these data indicate that these genes are inhibited by E2 in both basal and induced states. In the basal state, E2 reduces levels of AcH3. In the induced (by TNF-α) state, E2 reduces the levels of p65 and AcH3.

### E2, Through ERβ, Inhibits MCP-1 and CINC-2β mRNA Expression in TNF-α treated RASMCs

**Figure 7:**
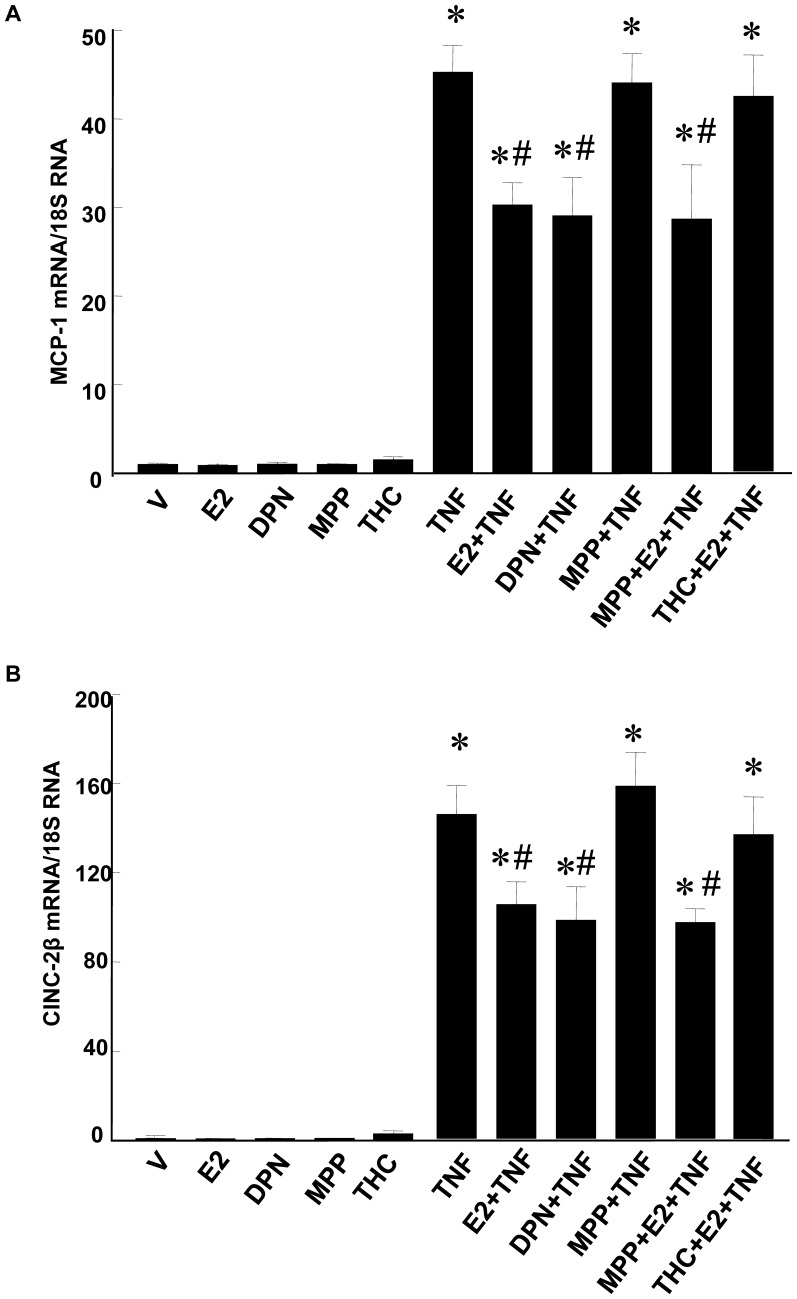
E2 inhibited TNF-α-induced MCP-1 and CINC-2β mRNA expression in RASMCs through ERβ. Cells were grown to subconfluence (≈95%) in 6-well plates, deprived of serum for 24 hrs, pretreated with E2 (10^−7^ M), DPN (10^−7^ M) or vehicle for 24 h, and then treated with TNF-α (1 ng/ml) for an additional 1 hr. MPP (10^−6^ M), or R, R-THC (10^–6^ M) was given to cells at 1h before E2 treatment in some experiments. Data, expressed as means±SEM, are from real-time quantitative RT-PCR assays and are normalized by 18 S RNA. Data for MCP-1 and CINC-2β are standardized to the mean mRNA level of the TNF-α-treated RASMCs. *p<0.05 vs. respective vehicle-treated RASMCs; #p<0.05 vs. respective TNF-α-treated RASMCs.

To test whether E2 inhibits TNF-α-induced MCP-1 and CINC-2β mRNA expression and to assess the ER subtype dependence of the E2 effect, RASMCS were pretreated with E2, the selective ERβ agonist DPN, the selective ERα antagonist MPP alone or the selective ERβ antagonist R,R-THC alone in combination with E2, or vehicle for 1 hr and subjected to real time RT-PCR analysis for MCP-1 and CINC-2β mRNA, respectively. Quantitative real time RT-PCR analysis showed that TNF-α stimulated expression of MCP-1 and CINC-2β significantly compared to the vehicle control (Figure 7). Pretreatment with E2 or DPN significantly inhibited expression of MCP-1 and CINC-2β in cells treated with TNF-α. In contrast, R,R-THC, but not MPP antagonized the inhibitory effects of E2 on MCP-1 and CINC-2β mRNA expression in TNF-α-treated cells. E2, DPN, MPP or R,R-THC alone did not alter MCP-1 and CINC-2β mRNA in RASMCs in the absence of TNF-α treatment. Together, findings suggest that the E2 mediated anti-inflammatory effect in TNF-α treated RASMCs is mediated by ERβ, and not ERα.

## Discussion

The multifaceted crosstalk between NFκB signaling and the ERs has been well documented [31]. In numerous models, E2 and ERs have been shown to increase levels of IκBα and reduce levels of phosphorylated IκBα [28], [32]–[34]. Moreover, both ERα and ERβ reportedly inhibit NFκB activity in an E2 dependent manner in a variety of cell types [31], [35]–[42], and molecular studies have mapped the minimal domains of ERα necessary for these effects to the ligand binding domain (LBD), hinge domain and DNA binding domain (DBD) [43], [44]. *In vitro*, ERα binds to NFκB p65, p50 and c-Rel [43], [45]; ERβ inhibits the DNA binding ability of NFκB p50, c-Rel and NFκB p65/p50 dimers [36], [43], [46], and both ERs can prevent NFκB from binding to the *IL-6* promoter [43], [46], [47]. However, at present, there is a paucity of data to clarify the role of E2 and/or ERs in regulating the activity of NFκB in vascular cells.

Previously, we demonstrated that isolated RASMCs express high levels of inflammatory mediators, including the neutrophil- and monocyte-selective chemokines CINC-2β and MCP-1, when stimulated by TNF-α and that E2 inhibits this process and reduces the neutrophil chemotactic activity of media conditioned by TNF-α treated RASMCs via an ERβ-dependent mechanism [16]. Herein we extend our studies in order to elucidate the molecular mechanisms by which E2 and ERβ negatively regulate the NFκB signaling pathway in RASMCs. Specifically, this study demonstrates for the first time the multifaceted effects of E2 in negatively modulating events in the NFκB pathway in a vascular cell type. We show that E2 neither inhibits the production of TNF-α by RASMCs (See Text S1 and Figure S1), nor blocks the nuclear translocation of NFκB p65 (Figure S2). Further, we demonstrate that both ERα and ERβ proteins are expressed in our RASMCs in an E2 and TNF-α independent manner (Figure S3). We demonstrate that E2, via ERβ, attenuates signaling through the NFκB signaling pathway via a novel bimodal mechanism. First, E2 selectively enhance NFκB p65 binding to the *IκBα* promoter in order to stimulate the expression of IκBα, a direct inhibitor of NFκB activation. Second, E2 reduces the ability of NFκB p65 to bind to the promoters of pro-inflammatory genes such as *MCP-1* and *CINC-2β*, thereby inhibiting their transcriptional activity, indicated by the binding of AcH3 to the promoters, and mRNA expression. These findings support the intriguing hypothesis that E2, via ERβ, selectively modulates the nuclear activity of NFκB p65 to ensure that NFκB signaling is dampened by heightened IκBα levels, as well as by reducing the binding of nuclear NFκB p65 to the promoters of genes that mediate the inflammatory response.

IκBα is the one of the best documented inhibitors and transcriptional targets of NFκB. Through its ability to interact with NFκB proteins, IκBα masks the DBD of NFκB in order to maintain NFκB inactive in the cytoplasm until such time that NFκB is activated. While NFκB is initially activated through proteasomal-mediated degradation of IκBα, NFκB signaling is ultimately terminated through NFκB mediated resynthesis of IκBα, which re-establishes the inactive cytoplasmic pool of NFκB/IκBα complexes [48], [49]. Studies of the murine *IκBα* promoter identified six NFκB and NFκB-like response elements that are highly conserved in sequence, orientation and position within the genomes of humans and pigs [48]. Although the *IκBα* promoter appears to be devoid of NFκB proteins in the basal state, the *IκBα* promoter is bound and activated by NFκB proteins within minutes of NFκB activation [50], [51].

Our studies demonstrate that neither DPN nor E2 when administered alone stimulated IκBα mRNA expression in RASMCs despite substantial recruitment of NFκB p65 at the IκBα promoter. Furthermore, E2 alone -induced recruitment of NFκB p65 was not accompanied by recruitment of AcH4 at the *IκBα* promoter, indicating that the increased p65 binding was insufficient to increase *IκBα* gene transcription. This finding suggests that other unidentified cofactors are required for NFκB p65-induced transcription of the *IκBα* gene under these conditions. However, when cells were pretreated with E2 or DPN and then challenged with TNF-α, both E2 and DPN further enhanced the TNF-α-induced increases in IκBα mRNA expression and protein levels, suggesting the possibility that TNF-α may have recruited cofactors needed for *IκBα* gene transcription. The binding of ERβ, but not ERα at the *IκBα* promoter was increased by E2 treatment. The ERβ antagonist R,R-THC blocked the enhancement effects of E2 on *IκBα* gene transcription (p65 and AcH4 binding) and expression (mRNA and protein), suggesting that E2 may inhibit NFκB signaling by specifically targeting and enhancing events at the *IκBα* promoter, perhaps in a manner dependent on ERβ. Curiously, using a computer program that analyzes promoters for putative transcription factor binding sites, we failed to identify any potential ER binding elements (ERE) within the *IκBα* promoter. These data suggest that ERβ may not interact directly with the *IκBα* promoter to promote the binding of NFκB p65 to the promoter, but instead may work through recruitment of cofactors that enhance both binding of NFκB p65 to the promoter and transcription of the *IκBα* gene. Future studies will address how ERβ is required for E2 mediated NFκB recruitment to and enhanced transcription of the *IκBα* gene.

In addition, we have observed that NFκB p65 is rapidly recruited to the *MCP-1* and *CINC-2β* promoters in the presence of TNF-α. Under these conditions, ERβ is absent from these promoters, and transcriptional activity of these genes is significantly increased compared to vehicle treatment, as indicated by AcH3 binding on these promoters and mRNA expression of these genes. In response to E2 pretreatment, binding of NFκB p65 to these promoters is greatly reduced and binding of ERβ is greatly increased, transcriptional activity of these genes is significantly reduced, as indicated by decreased binding of AcH3 on these promoters and mRNA expression of these genes. At present, we can not definitively state why binding of ERβ and NFκB p65 at the *MCP-1* and *CINC-2β* promoters is mutually exclusive. Using computer programs designed to identify putative ERE, we could not identify any EREs within either the *MCP-1* or *CINC-2β* promoters. Thus, these findings suggest that the presence of ERβ at these promoters may occur through the use of an element that remains to be identified, or that ERβ interacts with these promoters indirectly, i.e., through another DNA-binding protein (cofactor). Our future studies are attempting to address this question.

In summary, this study has elucidated a novel bimodal mechanism by which E2 inhibits NFκB signaling and thereby the inflammatory response to TNF-α in RASMCs. E2 both 1) enhances expression of IκBα, a direct inhibitor of NFκB activation, thus accelerating a negative feedback loop in NFκB signaling, and 2) directly inhibits binding of NFκB p65 to the promoters of inflammatory genes, including *MCP-1* and *CINC-2β*, thereby inhibiting their expression. The findings that, in the presence of E2+TNF-α, ERβ is recruited and the binding of NFκB is reduced at the *MCP-1* and *CINC-2β* promoters, suggest that the ability of selective ERβ activation to inhibit expression of inflammatory mediators in activated RASMCs may be related, in part, to interference with the DNA binding ability of NFκB p65 by ERβ.

## Methods

### Cell Culture

Primary cultures of RASMCs were derived from 10-week-old female Sprague-Dawley rats (Charles River), as previously described [16], [52]. All protocols were approved by the Institutional Animal Care and Use Committee of the University of Alabama at Birmingham and were consistent with the Public Health Service Policy on Humane Care and Use of Laboratory Animals (Office of Laboratory Animal Welfare, August 2002) and the Guide for the Care and Use of Laboratory Animals published by National Institutes of Health (NIH Publication No. 96-01, revised in 2002). The animal protocol number is 100908574. Cells were cultured in complete medium containing phenol red–free DMEM (Gibco) supplemented with 10% (vol/vol) FBS, 4 mmol/L L-glutamine, 100 U/mL penicillin, and 100 µg/ml streptomycin. RASMCs were pre-treated with E2 (10^−7^ M) or vehicle (ethanol at a final concentration <0.01%) for 24 hrs in all experiments. Cells were used within 5 passages and were identified as RASMCs by their characteristic morphology and positive immunostaining for α-smooth muscle actin (α-SMA, clone 1A4, DAKO). RASMCs pre-treated with or without E2 for 24 hours were then incubated with TNF-α (1 ng/mL) for various time periods from 10 min to 6 hrs. To assess the ER dependence of the E2 effect on IκBα expression, cells were pretreated with the selective ERβ agonist DPN (10^–7^ M) or the selective ERα agonist PPT (10^–7^ M) (Tocris Cookson, Ellisville, MO) for 24 hrs and then incubated with 1 ng/ml TNF-α for an additional 45 or 60 min. Another set of cells from the above experiments were exposed to the selective ERα antagonist MPP (10^−6^ M) or the selective ERβ antagonist R,R-THC (10^−6^ M) (Tocris Cookson, Ellisville, MO) for 1 hr before the E2 (10^–7^ M) pretreatment.

### Real-time Quantitative RT-PCR Analyses

Real-time quantitative RT-PCR analysis was performed as described before [9], [10], [16]. Total RNA was extracted from cells using TRIzol (Invitrogen, Carlsbad, CA), and treated with DNAase I to remove genomic DNA. The protein- and DNA-free RNA was reverse transcribed to cDNA and analyzed using the SYBR Green RT-PCR kit (Applied Biosystems, Foster City, CA) and specific primers: *IκBα* forward, 5′-CAGCAGACTCCACTCCACTT-3′ and *IκBα* reverse, 5′-GAGAGGGGTATTTCCTCGAA-3′. *MCP-1* forward 5′-ATGCAGGTCTCTGTCACGCT -3′ and *MCP-1* reverse, 5′-GGTGCTGAAGTCCTTAGGGT-3′; *CINC-2β* forward 5′- TCAGGGACTGTTGTGG -3′ and *CINC-2β* reverse, 5′- TGACTTCTGTCTGGGTG-3′. cDNA was amplified by PCR in the iCycler for 40 cycles and relative RNA levels were calculated using the iCycler software. Samples were compared by the relative (comparative) Ct method. Fold induction or repression was measured relative to controls and calculated after adjusting for 18 s RNA (endogenous control) using 2^−ΔΔCt^, where Δ Ct = Ct interested gene - Ct 18 s RNA and ΔΔCt = ΔCt treatment - ΔCt vehicle control.

### Immunoblot Analyses

Quiescent RASMCs were incubated with E2 or vehicle for 24 hrs, followed by TNF-α for 10, 20, 30, 40, 50 and 60 min. Total protein was extracted and total and phospho-IκBα levels were assessed using Western blot analysis with selective anti-IκBα (Santa Cruz) and anti-phospho-IκBα (Cell Signaling) antibodies. Expression of ERα and ERβ protein was assessed using Western blot analysis with selective anti-ERα (Santa Cruz HC-20) and anti-ERβ (Millipore 07-359) antibodies. Protein loading was assessed by stripping the membranes and reprobing with anti-β-actin antibody (Sigma).

### Chromatin Immunoprecipitation Assays

RASMCs were pretreated with E2 (10^−7^ M) or vehicle for 24 hrs and then treated with TNF-α (1 ng/mL) or vehicle for 1 hr. Cells were fixed with formaldehyde and subjected to chromatin immunoprecipitation (ChIP) analyses as previously described [53]–[55]. Briefly, cells were fixed with formaldehyde for 15 min and nuclei purified, then passed through a 22-gauge needle three times and sonicated to an average size of 500–1000 bps. Protein-DNA complexes were immunoprecipitated (IP) using 5 µg of antibodies selective for NFκB p65 (Abcam), ERβ (Millipore), AcH3 or AcH4 (Upstate Signaling Solutions). The immune complexes were adsorbed with protein A beads or protein G beads blocked with bovine serum albumin and salmon sperm DNA (Upstate Signaling Solutions). Immunoprecipitants were washed, eluted and crosslinks were reversed overnight. The next day, samples were digested with Proteinase K and clarified by phenol:chloroform:isoamyl alcohol extraction. DNA was purified using mini spin columns and IP and non-IP DNA (Input) was analyzed by real time PCR using specific primers: *IκBα* forward, 5′ AAGTCGTCGGTGGGAAAC 3′ and *IκBα* reverse, 5′ CCTGAGTGGCTGGAAAGT 3′ that amplify −405 to −280 in the rat *IκBα* gene promoter; *MCP-1* forward 5′ GCACTTACTCAGCAGATTC 3′ and reverse, 5′ GCCTCAGCCTTTTATTGT 3′ that amplify −208 to −91 in the rat *MCP-1* gene promoter; forward 5′ CAAACGAGGACTGGGTAG 3′ and reverse, 5′ GACTTAGGTGCAGGGACT 3′ that amplify −346 to −541 in the rat *CINC-2β* gene promoter. Results are representative of three experiments.

### Statistical Analysis

Data are expressed as mean±SEM. Statistical analysis was performed with one-way ANOVA or Student’s t test, as appropriate. Values of P<0.05 were considered significant.

## Supporting Information

Figure S1 **Co-treatment with E2 and TNF-α does not stimulate TNF-α expression in RASMCs.** Cells were grown to subconfluence (≈95%) in 6-well plates, deprived of serum for 24 hrs, pretreated with 10^−7^ M E2 or vehicle for 24 hrs, then treated with TNF-α (1 ng/mL) for the periods indicated. Conditioned media was collected. Data, expressed as means±SEM, are from a double sandwich ELISA assay.
(TIF)

Figure S2 **Representative micrographs of RASMCs pretreated with E2 (10^−7^ M) or vehicle for 24 hrs before incubated with TNF-α (1 ng/mL) for 30 min.** Cells were analyzed using anti-NFκB p65 antibody (A1,B1,C1,D1) and nuclei were stained with DAPI (A2,B2,C2,D2). Merged images are shown in the panel A3,B3,C3,D3. E. Bar graph demonstrating the percentage of cells with NFκB p65 nuclear translocation after TNF-α±E2 treatment for 0, 15, 30 and 60 min. Results are mean±SE from 3 slides/group; a total of >200 cells were counted/group). *P<0.05 compared with vehicle control group.
(TIF)

Figure S3 **Representative Western blots of ERα and ERβ in E2±TNF-α treated RASMCs.** Cells were pretreated with E2 (10^−^7 M) or vehicle for 24 h, and then treated with TNF-α (1 ng/ml) for an additional 6 hrs. Blots was reprobed with antibody against β-actin for input loading.
(TIF)

Text S1 **Detailed protocol.**
(DOC)
