# Supplementary figures and images for: Estrogen Modulates NFκB Signaling by Enhancing IκBα Levels and Blocking p65 Binding at the Promoters of Inflammatory Genes via Estrogen Receptor-β
Source: PLoS One. 2012 Jun 19;7(6):e36890. doi: 10.1371/journal.pone.0036890 (PMC3378567; doi:10.1371/journal.pone.0036890)

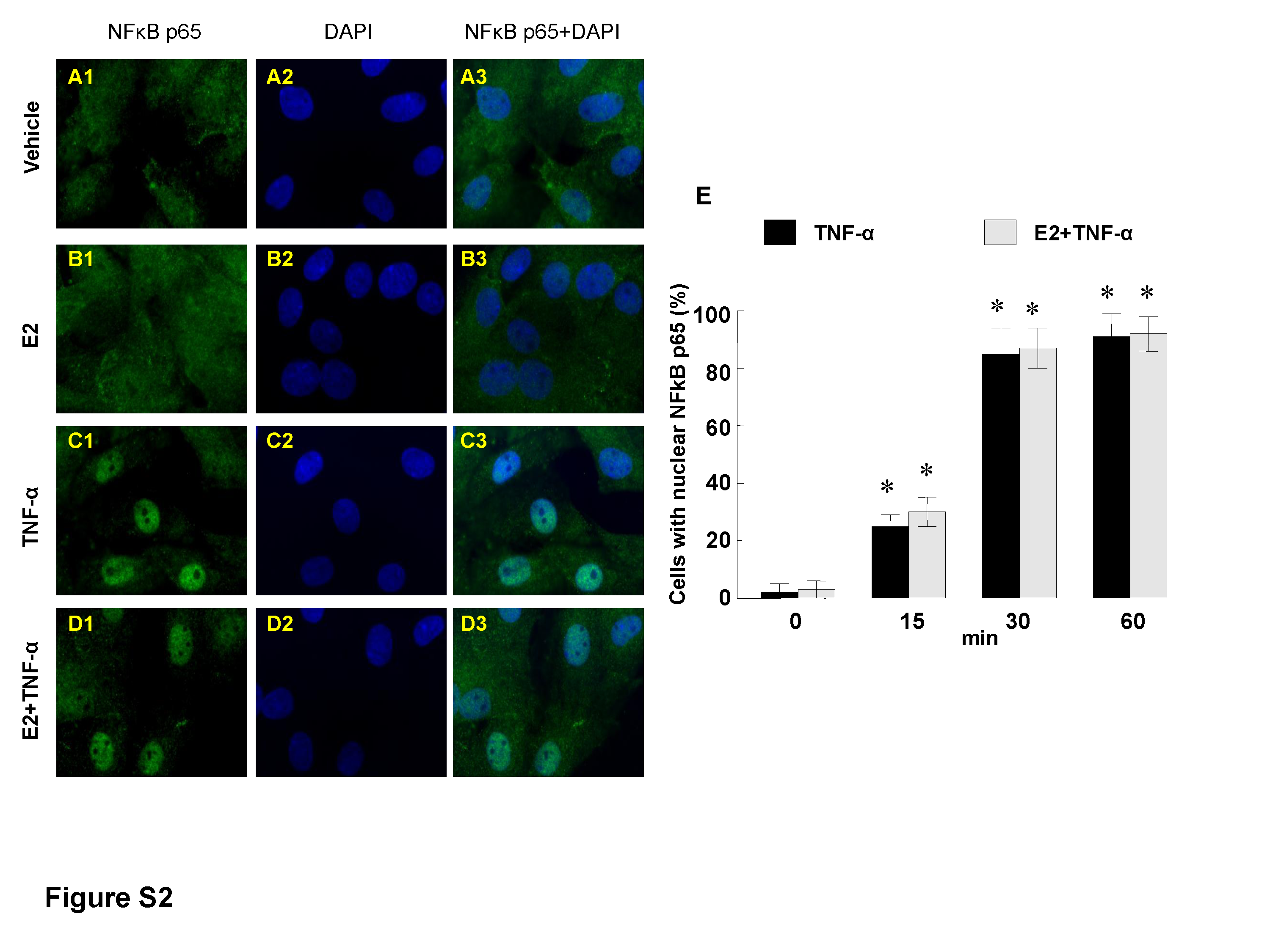

Supplement: Figure S2 — Representative micrographs of RASMCs pretreated with E2 (10−7 M) or vehicle for 24 hrs before incubated with TNF-α (1 ng/mL) for 30 min. Cells were analyzed using anti-NFκB p65 antibody (A1,B1,C1,D1) and nuclei were stained with DAPI (A2,B2,C2,D2). Merged images are shown in the panel A3,B3,C3,D3. E. Bar graph demonstrating the percentage of cells with NFκB p65 nuclear translocation after TNF-α±E2 treatment for 0, 15, 30 and 60 min. Results are mean±SE from 3 slides/group; a total of >200 cells were counted/group). *P<0.05 compared with vehicle control group. (TIF) [file pone.0036890.s002.tif]

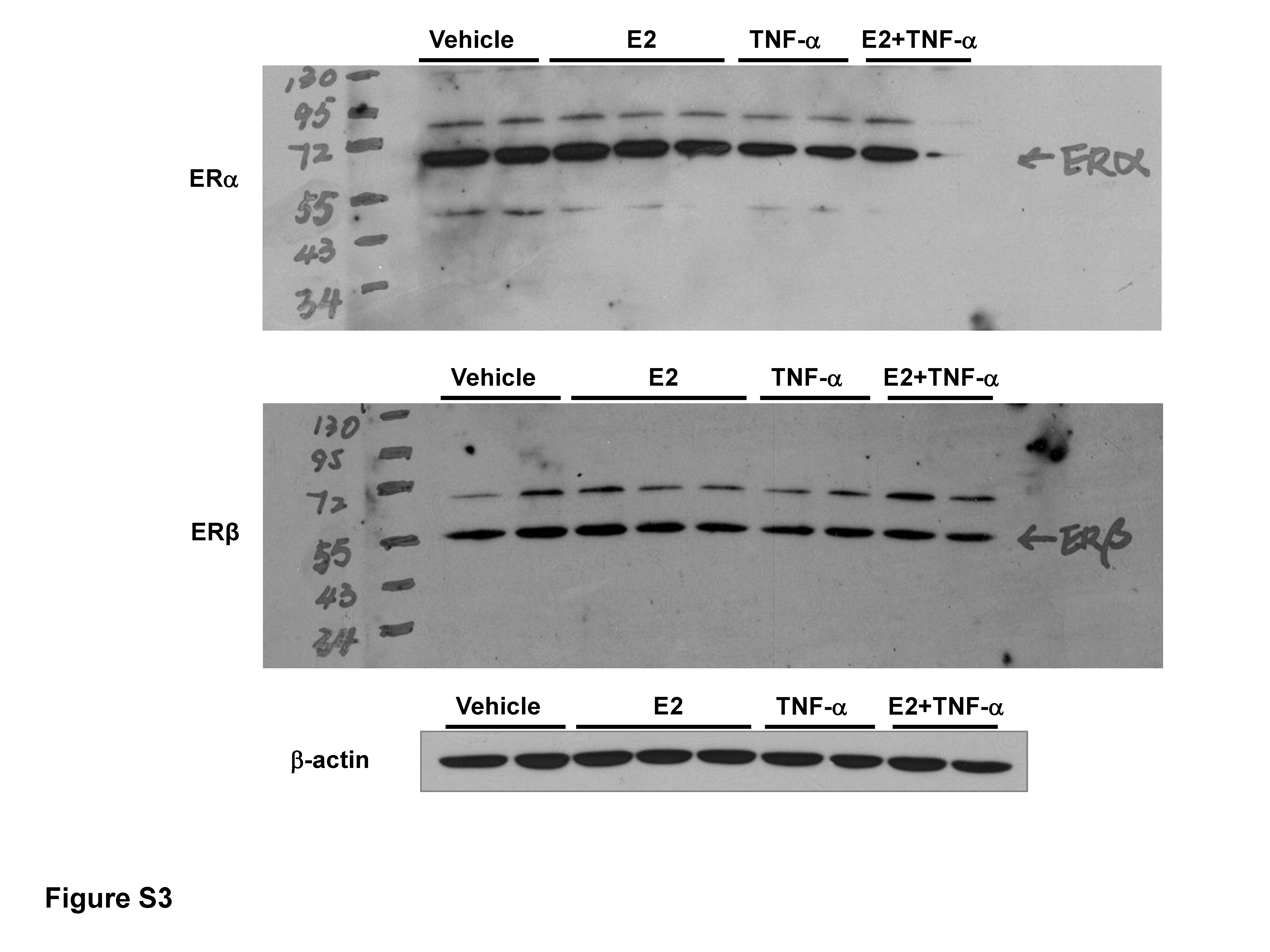

Supplement: Figure S3 — Representative Western blots of ERα and ERβ in E2±TNF-α treated RASMCs. Cells were pretreated with E2 (10−7 M) or vehicle for 24 h, and then treated with TNF-α (1 ng/ml) for an additional 6 hrs. Blots was reprobed with antibody against β-actin for input loading. (TIF) [file pone.0036890.s003.tif]
