# Supplementary material for: Estrogen Modulates NFκB Signaling by Enhancing IκBα Levels and Blocking p65 Binding at the Promoters of Inflammatory Genes via Estrogen Receptor-β
Source: PLoS One. 2012 Jun 19;7(6):e36890. doi: 10.1371/journal.pone.0036890 (PMC3378567; doi:10.1371/journal.pone.0036890)
Supplement: Text S1 — Detailed protocol. (DOC) [file pone.0036890.s004.doc]

**SUPPLEMENT**

**METHODS**

**ELISA**

RASMCs pre-treated with or without E2 for 24 hours were then incubated with TNF-α (1 ng/mL) for various time periods from 10 min to 6 hrs. Conditioned media (5 ml) was collected at each time point and assayed with the rat TNF-α ELISA kit (BD Biosciences Pharmingen) according to the manufacturer's instructions. The sensitivity of the kit is in the 31.3 - 2000 pg/ml range. A second set of RASMCs was pretreated with E2 (10^-7^ M) or vehicle for 24 hrs, followed by TNF-α (1 ng/mL) or vehicle for 15 min. At the 15 min time point, TNF-α was removed and replaced with fresh DMEM containing E2 or vehicle. Cells were incubated for periods of 1, 2, 3, 4, 5 and 6 hrs, as above, and conditioned media (5 ml) was collected for measurement of TNF-α by ELISA.

# Immunofluorescent Staining

RASMCs were fixed in 4% paraformaldehyde, permeabilized in 0.1% Triton X-100 in PBS, and stained with selective anti–NFκB p65 primary antibody (1:100, Santa Cruz Biotechnology) overnight at 4°C and with a FITC-conjugated goat anti-rabbit IgG secondary antibody (1:500, Vector Laboratories) for 1 hr at room temperature, then mounted with Vectashield mounting medium containing DAPI (Vector Laboratories) and covered with a coverslip. Two controls, in which we omitted the primary or secondary antibody, were included in each experiment. Samples were analyzed with an Olympus microscope with appropriate excitation/emission filter pairs.

**RESULTS**

**Co-treatment with E2 and TNF-α Does Not Stimulate TNF-α Expression in RASMCs**

E2 has been shown to downregulate the production and/or the release of TNF-α by several cell lineages, including monocytes and bone stromal cells [1-4]. To test the hypothesis that E2 inhibits the expression of endogenous TNF-α in TNF-α or vehicle treated RASMCs in culture, cells were pretreated with E2 or vehicle for 24 hrs, followed by TNF-α or vehicle for periods of 1, 2, 3, 4, 5, and 6 hrs. Total RNA was extracted and the levels of TNF-α mRNA were analyzed by real-time quantitative RT-PCR. TNF-α mRNA was undetectable in RASMCs treated with vehicle, E2, TNF-α or both (data not shown).

Levels of TNF-α protein measured by ELISA in conditioned media were in the range of 982-1047 pg/ml in all samples (Figure S1). TNF-α levels neither increased nor decreased during the 6 hr incubation period, suggesting that the TNF-α measured was exogenous and remained stable under culture conditions. When exogenous TNF-α was removed from the cells after 15 min incubation, TNF-α was undetectable in the conditioned media over the 1-6 hr incubation period, demonstrating that TNF-α was not synthesized and/or released by the cells (data not shown).

**E2 Does Not Inhibit TNF-α–Induced Nuclear Translocation of NFκB p65 in RASMCs**

To test whether E2 inhibits TNF-α Induced nuclear translocation of NFκB in RASMCs, quiescent cells were pretreated with E2 (10^-7^ M) or vehicle for 24 hrs, then incubated with TNF-α (1 ng/mL) or vehicle for an additional 15, 30 or 60 mins. In vehicle or E2 alone-treated cells, immunostaining of NFκB p65 was diffuse and distributed in both cytoplasm and nuclei. (Figure S2, A1-B1). TNF-α (1ng/mL) stimulated nuclear translocation of NFκB p65 (Figure S2, C1). This translocation occurred in a time-dependent manner, beginning at 15 min, peaking at 30 min and persisting until 60 min of treatment (Figure S2, E). Nearly 90% of TNF-α–treated RASMCs demonstrated nuclear localization of NFκB p65 (Figure S2, E). Pretreatment with E2 (10^-7^ M for 24 hrs) did not inhibit TNF-α–induced NFκB p65 translocation (Figure S2, D1).

**Neither E2 nor TNF-α Alters Protein Expression of ERα and ERβ in RASMCs**

To test whether E2 or TNF-α alters protein expression of ERα and ERβ in RASMCs, quiescent cells were pretreated with E2 (10^-7^ M) or vehicle for 24 hrs, then incubated with TNF-α (1 ng/mL) or vehicle for an additional 6 hrs. Whole cell lysate was collected and analyzed by Western blot with the selective antibodies against ERα and ERβ, respectively (Figure S3). Both ERα and ERβ protein were expressed in our RASMCs in an E2/TNF-α-independent manner.

**REFERENCE**

1. **Ralston SH** 1994 Analysis of gene expression in human bone biopsies by polymerase chain reaction: evidence for enhanced cytokine expression in postmenopausal osteoporosis. J Bone Miner Res 9:883-890.

2. **Rogers A, Eastell R** 2001 The effect of 17beta-estradiol on production of cytokines in cultures of peripheral blood. Bone 29:30-34.

3. **Ralston SH, Russell RGG, Gowen M** 1990 Estrogen inhibits release of tumor necrosis factor from peripheral blood mononuclear cells in postmenopausal women. J Bone Miner Res 5:983-988.

4. **Kimble RB, Srivastava S, Ross FP, Matayoshi A, Pacifici R** 1996 Estrogen deficiency increases the ability of stromal cells to support osteoclastogenesis via an IL-1 and TNF mediated stimulation of M-CSF production. J Biol Chem 271:28890-28897.
